# Frequency and factors associated with proteinuria in COVID-19 patients: a cross-sectional study

**DOI:** 10.11604/pamj.2021.40.37.29796

**Published:** 2021-09-14

**Authors:** Yannick Mayamba Nlandu, Theodore-Junior Manyka Sakaji, Yannick Mompango Engole, Pitchouna Marie-France Ingole Mboliasa, Dauphin Mulumba Bena, Jessy Mukamamvula Abatha, Jean-Robert Mpoke Nkumu, Aliocha Natuhoyila Nkodila, Karel Van Eckout, Golan Kalifa, Rodolphe Ahmed, Justine Busanga Bukabau

**Affiliations:** 1 Intensive Care Unit, Kinshasa Medical Center, Kinshasa, Democratic Republic of the Congo,; 2 Nephrology Unit, Kinshasa University Hospital, Kinshasa, Democratic Republic of the Congo,; 3 Faculty of Public Health, Lomo University of Research, Kinshasa, Democratic Republic of the Congo

**Keywords:** Proteinuria, COVID-19, Black African, oxidative stress, inflammation

## Abstract

Proteinuria is a marker of severity and poor outcome of patients in intensive care unit (ICU). The objective of this study was to determine the frequency of proteinuria and the risk factors associated with proteinuria in Congolese COVID-19 patients. The present cross sectional study of proteinuria status is a post hoc analysis of data from 80 COVID-19 patients admitted at Kinshasa Medical Center (KMC) from March 10^th^ to July 10^th^, 2020. The population under study came from all adult inpatients (≥18 years old) with a laboratory diagnosis by polymerase chain reaction (PCR) of COVID-19 were selected and divided into two groups (positive proteinuria and negative proteinuria group). Logistic regression models helped to identify the factors associated with proteinuria. The P value significance level was 0.05. Among 80 patients who tested positive for SARS-CoV-2 RT-PCR, 55% had proteinuria. The mean age was 55.2 ± 12.8 years. Fourty-seven patients (58.8%) had history of hypertension and 26 patients (32.5%) diabetes. Multivariable analysis showed age ≥ 65 years (aOR 5,04; 95% CI: 1.51-16.78), diabetes (aOR 3,15; 95% CI: 1.14-8.72), ASAT >40 UI/L (aOR 7,08; 95% CI: 2.40-20.87), ferritin >300 (aOR 13,47; 95% CI: 1.56-26.25) as factors independently associated with proteinuria in COVID-19 patients. Proteinuria is common in Congolese COVID-19 patients and is associated with age, diabetes, ferritin and aspartate aminotransferase (ASAT).

## Introduction

Since the coronavirus disease 2019 (COVID-19) was declared to be a pandemic, several studies have been carried out with the aim to pinpoint the risk factors associated with COVID-19-related mortality and thus, stratify the patients according to the severity of the disease. Beyond the fact of being a marker of kidney damage, proteinuria appear to be also a severity marker of COVID-19 [1]. A study describing urinalysis clinical significance for predicting COVID-19 severity, namely, a research carried on a group of 119 Chinese patients revealed that urine parameters facilitated COVID-19 identification and helped to determine dynamic changes in patients with COVID-19 [1].

Proteinuria is an increased amount of protein in the urine. It´s typically a marker for kidney or cardiovascular disease, a risk factor for kidney disease progression, and/or a manifestation of systemic disease in the kidney [2-4]. Proteinuria, in intensive care unit (ICU), is considered as a transient situation. It is also reported to be a disease severity marker and a mortality risk cause. Indeed, proteinuria was reported to be an independent predictor of all-cause mortality particularly in cirrhotic patients and in acute myocardial infarction patients [3-5]. Similar results was reported in ICU COVID-19 patients [1].

To date, some studies assess the burden of proteinuria in COVID-19 patients from sub-Saharan Africa (SSA). Nlandu *et al*. in Congolese COVID-19 patients revealed that proteinuria is a predictor of mortality [6]. In this context, the non-expensive dipstick proteinuria test must be an alternative for stratification of COVID-19 patients and the knowledge of the factors explaining this proteinuria could be used to reduce the COVID-19 mortality. The main objective of this study was to determine in hospitalized COVID-19 patients with moderate to severe disease, the frequency of proteinuria as well the risk factors associated with it.

## Methods

**Study design and participants:** the present cross sectional study is a post hoc analysis of data from COVID-19 patients enrolled from March 10^th^ to July 10^th^, 2020 [6]. Patients under study came from Kinshasa Medical Centre (KMC), a private hospital officially devoted to COVID-19 patients´ care. The inclusion criteria were strictly based upon laboratory confirmation of SARS-CoV-2 by qualitative reverse-transcriptase polymerase chain reaction (RT-PCR) test of nasopharyngeal swabs and the availability of proteinuria test. Patients under the age of 18 years old, with end-stage kidney disease (i.e. kidney transplantation or chronic dialysis), urinary tract infection (positive urine culture) and those with haematuria in urinalysis were ruled out.

**Data and collection:** the KMC electronic patient database enabled the manual extraction of clinical data. After patients were admitted to KMC, approximately 20 mL of clean midstream urine samples were collected from the subjects at admission. Urine sample collected from catheter was excluded. Urine proteinuria were visually evaluated with urine test kits (Combur 10 test-M, Cobas-Roche Technology, Switzerland) immediately after urine collection. All data were gathered at admission. We collected information demographic features; the presence of any chronic illness; vital signs; and laboratory tests. The description of study (data and collection) have already been presented [6].

**Definitions:** proteinuria was defined by presence of ≥1+ protein on urinalysis. Nephrotic-range proteinuria was defined by presence of ≥3+ protein on urinalysis. On admission in the emergency quarter, two criteria were used to classify patients, namely, as having proteinuria or not, depending on the presence of the aforesaid results or not. Hypertension was recorded if the patient was taking any antihypertensive drug or had two separate BP measurements ≥140/90 mmHg [7]. The diabetes diagnosis was based on diagnostic criteria from the American Diabetes Association as a presence of a fasting plasma glucose level of >126 mg/dl or usage of antidiabetic drugs diabetes [8]. Acute respiratory distress syndrome (ARDS) were defined according the Berlin definition respectively [9].

**Statistical analysis:** analyses were performed on SPSS 21.0. Descriptive statistics consisted of calculating the mean and standard deviation for quantitative data with Gaussian distribution; the median and interquartile range for quantitative data with non-Gaussian distribution. Proportions were used for categorical data. Pearson's chi-square test or Fisher's exact test was used to compare the proportions, while the student's t-test compared the means and Man Whitney's U-test made it possible to compare the medians. The search for factors associated with proteinuria was carried out by the logistic regression test in univariate analysis. When differences were observed between proteinuria and the independent variables, the effect of potential confounders was studied by logistic regression adjustment in multivariate analysis. Finally, the odds ratio (ORs) and their 95% confidence intervals (95%CI) were calculated to determine the degree of association between proteinuria and the independent variables. A p-value <0.05 was considered to be the threshold of statistical significance.

**Ethical considerations:** the study showed consideration for the rules of ethics, confidentiality and patients´ privacy. The examined files were recorded anonymously. The information collected during the history and clinical examination respected the confidentiality and privacy of patients. They were all transcribed into a pre-established and pre-coded investigation sheets. The National Ethics committee of Health, Democratic Republic of Congo (N °225/CNES/BN/PMMF/2020) authorized the present research projects on COVID-19. Written, informed was waived by the National Ethics committee of Health, Democratic Republic of Congo because of the urgency and unprecedented nature of the COVID-19 pandemic.

## Results

Among 80 patients who tested positive for SARS-CoV-2 RT-PCR, 44 (55%) had proteinuria. Among these 44 patients, 7 (15.9%) had a nephrotic range of proteinuria. Clinical and laboratory data of patients with proteinuria at admission are reported in Table 1 and Table 2. The mean age was 55.2 ± 12.8 years, with 47 patients (58.8%) with a history of hypertension and 26 patients (32.5%) with diabetes. Patients with proteinuria at admission were older and had a higher prevalence of hypertension, diabetes, fever as symptom, respiratory rate more than 24 cycles per minute (Table 1), aspartate amino transferase (ASAT) >40 UI/L, ferritin >300 ng/mL, procalcitonin (PCT) ≥0.5 ng/mL, C-reactive protein (CRP) >50 mg/L, brain natriuretic peptide (BNP) >300 pg/mL, troponin >28 ng/mL.

**Table 1:** clinical characteristics according to proteinuria status of 80 COVID-19 patients admitted to the Kinshasa Medical Center (KMC) from March 10^th^ to July 10^th^, 2020

	Over all (n=80)	Negative proteinuria (n=36)	Positive proteinuria (n=44)	p
Age (years)	55.2 ± 12.8	49.6 ± 12.9	59.7 ± 10.9	**<0.001**
Age ≥65 years	21 (26.3)	4 (11.1)	17 (38.6)	**0.005**
**Sex**				0.097
Male	60 (75.0)	24 (66.7)	36 (81.8)	
Female	20 (25.0)	12 (33.3)	8 (18.2)	
Hypertension (yes)	47 (58.8)	16 (44.4)	31 (70.5)	**0.017**
Diabetes (yes)	26 (32.5)	7 (19.4)	19 (43.2)	**0.021**
Heart failure (yes)	5 (6.3)	2 (5.6)	3 (6.8)	0.596
Fever (yes)	56 (70.0)	19 (52.8)	37 (84.1)	**0.002**
Cough (yes)	49 (61.3)	19 (52.8)	30 (68.2)	0.120
Dyspnea (yes)	32 (40.0)	15 (41.7)	17 (38.6)	0.481
Weakness (yes)	31 (38.8)	11 (30.6)	20 (45.5)	0.129
Anorexia (yes)	7 (8.8)	1 (2.8)	6 (13.6)	0.092
Headache (yes)	10 (12.5)	2 (5.6)	8 (18.2)	0.085
Vomissements (yes)	3 (3.8)	1 (2.8)	2 (4.5)	0.576
SBP, mmHg	140.6 ± 18.1	137.2 ± 18.4	142.9 ± 17.7	0.215
SBP >140 mmHg	28 (43.1)	8 (30.8)	20 (51.3)	0.083
DBP, mmHg	86.0 ± 13.5	85.6 ± 14.8	86.3 ± 12.8	0.839
HR, bpm	92.7 ± 14.7	86.9 ± 14.2	96.6 ± 13.8	**0.008**
RR, cycle/min	25.7 ± 8.5	23.7 ± 8.7	26.7 ± 8.3	0.211
RR >24 cpm	22 (39.3)	4 (21.1)	18 (48.6)	**0.041**
BOS, %	94.4 ± 6.9	93.9 ± 7.5	94.7 ± 6.6	0.682
T, °C	37.4 ± 0.94	37.2 ± 0.7	37.6 ± 1.04	0.066
T ≥38°c	20 (31.3)	5 (19.2)	15 (39.5)	0.073
FiO2	0.91 ± 0.5	0.58 ± 0.31	1.04 ± 0.7	0.329
ARDS severity				0.788
No ARDS	6 (12.5)	3 (18.8)	3 (9.4)	
Mild ARDS	7 (14.6)	2 (12.5)	5 (15.6)	
Moderate ARDS	11 (22.9)	4 (25.0)	7 (21.9)	
Severe ARDS	24 (50.0)	7 (43.8)	16 (53.1)	

Data are expressed as mean ± standard, n (%), or n/N (%); p values were calculated by Mann-Whitney U test, χ2 test, or Fisher's exact test, as appropriate; ARDS: acute respiratory distress syndrome; Bpm: beats per minutes; BOS: blood oxygen saturation; CKD: chronic kidney disease; DBP: diastolic blood pressure; FiO2: fraction inspired of oxygen; HR: heart rate; RR: respiratory rate; p-values written in bold indicate significant p-values (<0.05)

**Table 2:** biological characteristics according to proteinuria status of 80 COVID-19 patients admitted to the Kinshasa Medical Center (KMC) from March 10^th^ to July 10^th^, 2020

	Over all (n=80)	Negative proteinuria (n=36)	Positive proteinuria (n=44)	p
ASAT. UI/L	51.5 (28.0 - 90.8)	33.0 (22.0 - 70.5)	73.0 (47.0 - 103.8)	**0.001**
ASAT>40 UI/L	55 (68.8)	17 (47.2)	38 (86.4)	**<0.001**
ALAT UI/L	39.5 (25.0 - 68.8)	32.0 (20.0 - 65.8)	46.5 (29.3 - 68.8)	0.490
ALAT>40 UI/L	39 (48.8)	14 (38.9)	25 (56.8)	0.085
Ferritin. ng/mL	1200.0 (691.9 - 1200.0)	1200.0 (248.8 - 1200.0)	1200.0 (953.9 - 1200.0)	0.907
Ferritin >300 ng§mL	51 (85.0)	19 (70.4)	32 (97.0)	**0.005**
LDH >245 UI/L	55 (70.5)	23 (67.6)	32 (72.7)	0.405
Troponin. ng/L	9.2 (3.2 - 26.9)	5.4 (1.8 - 14.4)	13.1 (3.6 - 50.1)	0.120
Troponin >28 ng/L	16 (22.2)	3 (10.0)	13 (31.0)	**0.032**
PTr. %	73.4 ± 15.9	69.9 ± 16.8	75.9 ± 14.8	0.116
PTr <70%	27 (38.0)	15 (50.0)	12 (29.3)	0.063
PCT. ng/mL	0.2 (0.09 - 0.66)	0.12 (0.05 - 0.49)	0.34 (0.14 - 0.91)	0.413
PCT ≥0.5 ng/mL	26 (32.9)	8 (22.9)	18 (40.9)	**0.005**
CK. UI/L	173.0 (87.5 - 376.0)	138.0 (80.0 - 251.3)	255.0 (100.0 - 547.0)	0.105
CK >185 UI/L	34 (46.6)	12 (37.5)	22 (53.7)	0.128
CRP. mg/L	131.5 (55.8 - 218.0)	90.5 (23.0 - 216.5)	148.5 (92.3 - 241.8)	0.099
CRP >50 mg/L	63 (78.8)	23 (63.9)	40 (90.9)	**0.004**
Creatinine. μmol/L	83.5 (73.3 - 103.8)	81.0 (72.3 - 96.0)	94.5 (74.0 - 118.8)	**0.025**
Creatinin >133μmol/L	7 (8.8)	1 (2.8)	6 (13.6)	0.092
ProBNP >300 pg/mL	27 (35.5)	7 (21.2)	20 (46.5)	**0.020**
Glycemia. mg/dL	126.0 (102.3 - 179.0)	109.0 (97.5 - 145.3)	136.5 (103.8 - 183.8)	0.869
Hb. g/dL	13.2 ± 2.2	13.4 ± 2.1	13.1 ± 2.2	0.543
Ht. %	37.9 ± 7.0	39.0 ± 5.8	37.0 ± 7.9	0.227
WBC count. x 109	7.1 ± 3.2	7.0 ± 4.1	7.2 ± 2.4	0.793
Fibrinogen. g/L	6.9 ± 2.2	6.9 ± 2.3	7.0 ± 2.2	0.733
ProBNP. pg/mL	127.0 (46.5 - 651.3)	91.0 (43.5 - 245.9)	293.0 (45.0 - 1081)	0.301
paO2. mmHg	86.8 ± 35.6	93.2 ± 34.9	81.9 ± 35.7	0.188
paCO2. mmHg	34.0 ± 6.8	34.0 ± 5.7	34.0 ± 7.5	0.990
SaO2. %	94.8 ± 4.7	95.8 ± 4.3	94.0 ± 4.8	0.115
HCO3. mmol/L	23.6 ± 4.0	23.5 ± 3.6	23.7 ± 4.3	0.854
HbA1c. %	8.3 ± 2.8	8.9 ± 3.2	8.0 ± 2.6	0.423
TC. mmol/L	4.6 ± 1.5	4.5 ± 1.3	4.7 ± 1.7	0.789
LDLc. mmol/L	3.1 ± 1.4	3.2 ± 1.3	3.0 ± 1.5	0.724
HDLc. mmol/L	0.98 ± 0.62	1.02 ± 0.88	0.94 ± 0.34	0.722
Triglycerids. mmol/L	1.71 ± 1.0	1.43 ± 0.62	1.91 ± 1.22	0.188
LDH. UI/L	410.0 (232.0 - 656.3)	342.5 (221.3 - 523.5)	470.5 (237.0 - 814.5)	**0.031**
Total bilirubin. μmol/L	8.8 (6.1 - 12.5)	7.8 (5.6 - 10.8)	9.7 (6.3 - 13.9)	0.265

Data are mean ± standard, median (IQR), n (%), or n/N (%); p values were calculated by Mann-Whitney U test, χ2 test, or Fisher's exact test, as appropriate; ALAT: alanine amino transferase; ASAT: aspartate amino transferase; CRP: C reactive protein; Hb: hemoglobin; HDLc: high density lipoprotein cholesterol; LDH: lactate dehydrogenase; CK: creatinine kinase; pro BNP: brain natriuretic peptide; PTr: prothrombine time ratio; PCT: procalcitonin; TC: total cholesterol; WBC: white blood cell; p-values written in bold indicate significant p-values (<0.05)

The two groups comparison revealed that the group of patients with proteinuria had significantly higher age (59.7 ± 10.9 vs 49.6 ± 12.9), heart rate (96.6 ± 13.8 vs 86.9 ± 14.2), ASAT ( 73.0 (47.0-103.8) vs 33.0 (20.0-70.5)), creatinine (94.5 (74.0-103.8)) vs 81.0 (72.3-96.0)), lactate dehydrogenase (LDH) (470.5 (237.0-814.5)) vs 342.5 (221.3-523.5)) (Table 2). In multivariate analysis, age ≥65 years (aOR 5,04; 95% CI: 1.51-16.78), diabetes mellitus (aOR 3,15; 95% CI : 1.14-8.72), ASAT >40 UI/L (aOR 7,08; 95% CI: 2.40-20.87), ferritin >300 ng/mL (aOR13,47; 95% CI: 1.56-26.25) were the independent factors associated with proteinuria in COVID-19 patients (Table 3).

**Table 3:** factors associated with proteinuria in 80 COVID-19 patients admitted to the Kinshasa Medical Center (KMC) from March 10^th^ to July 10^th^, 2020

Variables	p	Unadjusted OR (IC95%)	p	Adjusted OR (IC95%)
Age ≥65 years				
No		1		1
Yes	0.008	5.04 (1.51 - 16.78)	**0.008**	5.04 (1.51 - 16.78)
Hypertension				
No		1		1
Yes	0.020	2.98 (1.19 - 7.50)	0.967	1.03 (0.24 - 4.50)
Diabetes mellitus				
No		1		1
Yes	0.027	3.15 (1.14 - 8.72)	**0.006**	6.38 (1.96 - 12.68)
ASAT >40 UI/L				
No		1		1
Yes	<0.001	7.08 (2.40 - 20.87)	**0.032**	8.48 (2.29 - 14.70)
Ferritin >300 ng/mL				
No		1		1
Yes	0.018	13.47 (1.56 - 26.25)	**0.017**	6.80 (2.45 - 10.69)
CRP >50 mg/L				
No		1		1
Yes	0.006	5.65 (1.65 - 19.39)	0.652	1.77 (0.15 - 2.20)
BNP >300 pg/mL				
No		1		1
Yes	0.025	3.23 (1.16 - 9.02)	0.896	1.11 (0.24 - 5.17)

P-values were calculated by logistic regression test; P-values written in bold indicate significant p-values (<0.05); ASAT: aspartate amino-transferase; CRP: C reactive protein; BNP: brain natriuretic peptide

## Discussion

In this cross-sectional study including 80 patients, the main results are that proteinuria is common in Congolese COVID-19 patients. Factors associated with proteinuria are age, diabetes mellitus, as well as oxidative stress markers (ferritin and AST). Many studies report a significant frequency of proteinuria during COVID-19. Our percentage of 55%, although similar to some studies [10,11], was twice that reported by Liu *et al*. [1] and far lower than 89.9% in the Bonetti *et al*. series [12]. This disparity could be explained on the one hand by differences in methodology between different studies, some not excluding diabetic, hypertensive and/or patients with chronic kidney disease and on the other hand by the characteristics of severe or non-severe study populations. Pei *et al*. for example, reported a frequency of 65.8% which rose to 85% when considering only critical cases of COVID-19 [13].

Cytokine storm causes kidney damage which could be responsible for proteinuria [1] and this proteinuria is mainly tubular. Huart *et al*. in a qualitative analysis of proteinuria during COVID-19, reported 1α-microglobulin as the main urinary protein [14]. Autopsy mainly reports tubular lesions in the COVID-19 patient [15] although a few non-tubular lesions have been reported [16]. In our study, only 15.9% of patients with proteinuria have a nephrotic range of proteinuria and can be considered to be suggestive of glomerular lesions. Indeed, the correlation between spot urinary protein creatinine ratio and dipstick for the diagnosis of nephrotic range of proteinuria was reported [17].

As in other studies involving severe non-COVID ICU patients [18], age, diabetes as well as markers of inflammation and/or oxidative stress (ferritin and ASAT) have been associated with proteinuria in our series. When it comes to age, there are on the one hand age related nephron structural changes, called nephrosclerosis that are responsible for proteinuria [19], and on the other hand, a high prevalence of comorbidities (arterial hypertension, diabetes mellitus and chronic renal disease) leading to proteinuria [19]. Proteinuria in diabetes mellitus has been described for several decades and is based on metabolic (glucose, AGE, polyols), hemodynamic (RAS) abnormalities as well as the production of reactive oxygen species responsible for functional and structural changes [20]. If there is no direct link between ASAT/ferritin and proteinuria, this association found in our study could be due to the relationship between oxidative stress associated with cytokine storm and proteinuria. Mortality prediction researches grounded on the ASAT/ALAT ratio testify that a ratio >1 may reveal systemic alterations, beyond liver sicknesses, as well as dysfunction at the mitochondrial level, causing or indicating an increased oxidative stress [21].

Ferritin, well known as inflammatory marker, is a useful marker that reflects the importance of oxidative stress [22]. In the course of infection, increased ferritin levels indicate a significant host defense mechanism depriving iron bacterial growth and safeguarding immune cell function. It possibly will also be protective, reducing the production of free radicals and mediating immunomodulation [23]. In short, proteinuria during COVID-19 would then be the reflection of this oxidative stress. Itokua *et al*.reported that albuminuiria is associated with decreased antioxidant capacity and increased levels of markers of hemolysis and inflammation in Congolese steady state children with sickle cell anemia [24].

Although the study was the first to examine proteinuria in the DRC, it, unfortunately, shows limitations worth to be mentioned. First, the results cannot be generalized to all COVID-19 patients simply because the study was undertaken in one single center. Second, the data related to the parameters of interest were not all easy to get in the context of a retrospective study. Third, the identification of potential associations between variables of interest could not be accurate due to the small sample size of the study. Four, distinguishing patients who had pre-existing proteinuria prior to admission at hospital was a big issue. Five, the use of semi-quantitative method to measure proteinuria.

## Conclusion

Proteinuria is common in Congolese COVID-19 patients and is associated with age, diabetes, ferritin and ASAT.

### What is known about this topic

- Proteinuria is a reported feature of COVID-19 and its frequency varies widely;
- Proteinuria is a severity marker of COVID-19.

### What this study adds

- Proteinuria is common in Congolese COVID-19 patients and nephrotic range proteinuria suggestive of glomerular lesions represent 15.9% of the patients with proteinuria at Kinshasa Medical Center;
- Oxidative stress and inflammatory markers are associated to proteinuria.
